# α-Adrenoceptor blockers and phaeochromocytoma surgery: outdated combination?^[Author-notes znac201-FM1]^

**DOI:** 10.1093/bjs/znac201

**Published:** 2022-06-16

**Authors:** Lisa Gunnesson, Maria Nilsson, Peter Larsson, Oskar Ragnarsson, Andreas Muth

**Affiliations:** Department of Surgery, Sahlgrenska University Hospital, Gothenburg, Sweden; Department of Surgery, Institute of Clinical Sciences, Sahlgrenska Academy, University of Gothenburg, Gothenburg, Sweden; Department of Surgery, Sahlgrenska University Hospital, Gothenburg, Sweden; Department of Anaesthesiology and Intensive Care Medicine, Sahlgrenska University Hospital, Gothenburg, Sweden; Department of Anaesthesiology and Intensive Care Medicine, Institute of Clinical Sciences, Sahlgrenska University hospital, Gothenburg, Sweden; Department of Endocrinology, Sahlgrenska University Hospital, Gothenburg, Sweden; Department of Internal Medicine and Clinical Nutrition, Institute of Medicine, Sahlgrenska Academy, University of Gothenburg, Gothenburg, Sweden; Department of Surgery, Sahlgrenska University Hospital, Gothenburg, Sweden; Department of Surgery, Institute of Clinical Sciences, Sahlgrenska Academy, University of Gothenburg, Gothenburg, Sweden

*Dear Editor*

Although recommended by current guidelines^1^, the use of preoperative α-adrenoceptor blockade (AAB) to prevent cardiovascular complications during phaeochromocytoma surgery continues to be debated^2^. Proponents of pretreatment see the widespread adoption of AAB in the 1960s^3^ as instrumental in improving surgical results. Opponents of pretreatment, on the other hand, argue that improved diagnostics with increased detection of phaeochromocytoma as adrenal incidentalomas, the advent of minimally invasive surgery, and improved anaesthetic practices have contributed to making contemporary phaeochromocytoma surgery safe. Indeed, a review^4^ of contemporary management from 21 centres worldwide published in this journal showed varying perioperative practices, and similar results with and without AAB, with an overall cardiovascular complication rate of 5 per cent and mortality rate of 0.5 per cent. However, the evidence base is weak and prospective data are lacking.

In this pilot study conducted at Sahlgrenska University Hospital, Gothenburg, Sweden, the feasibility of phaeochromocytoma surgery without AAB was investigated using a structured prospective protocol. All consecutive patients with newly diagnosed phaeochromocytoma between May 2017 and December 2019 were assessed for eligibility. Inclusion criteria were: newly diagnosed unilateral catecholamine-producing adrenal tumour, age over 18 years, and intended minimally invasive surgery. Exclusion criteria were: haemodynamic instability requiring intensive care, malignant hypertension (severe hypertension with secondary organ damage), symptomatic impaired cardiac function (New York Heart Association class III–IV), and pregnancy. The primary outcome variable was perioperative haemodynamics, defined as number of episodes of systolic BP over 200 or below 70 mmHg, monitored via arterial catheters and registered at least every 5 min. Secondary outcome variables were episodes of SBP over 180 or below 100 mmHg, time needed for surveillance in the postoperative ICU, use of inotropic agents when postoperative mean arterial pressure (MAP) reached levels below 65 mmHg, and total duration of inpatient stay. The study was approved by the regional ethical review board in Gothenburg (754-15). Informed consent was signed by all patients in the study group, but was not required for the control group as these patients were included retrospectively.

Of 15 patients available for assessment, 5 were excluded (AAB started before referral, 3; malignant hypertension, 1; bilateral tumours, 1). Ten patients diagnosed with phaeochromocytoma between 2017 and 2019 (4 men and 6 women; mean age 55 years) were therefore included and underwent minimally invasive adrenalectomy without AAB as soon as possible after inclusion. Surgery was performed under general sevoflurane-based anaesthesia aiming to maintain MAP over 65 mm Hg, systolic BP below 180 mm Hg, and heart rate under 100 b.p.m. These patients were compared with 10 age-matched controls from the authors’ institutional phaeochromocytoma database (240 patients) who had received traditional preoperative AAB treatment between 2003 and 2018 (*Table S1*).

There was no significant difference in perioperative or postoperative haemodynamics between the groups (*Fig. 1a*). Nine patients in the study group and only 2 in the control group received vasodilator infusions during surgery (*Table S1*). There was no significant difference between the study group and the control group with regard to tumour size or hormone profile (*Fig. 1b*). Patients without preoperative AAB spent significantly less time in postoperative surveillance immediately after surgery (*Fig. 1c*), as well as in hospital overall.

**Fig. 1:**
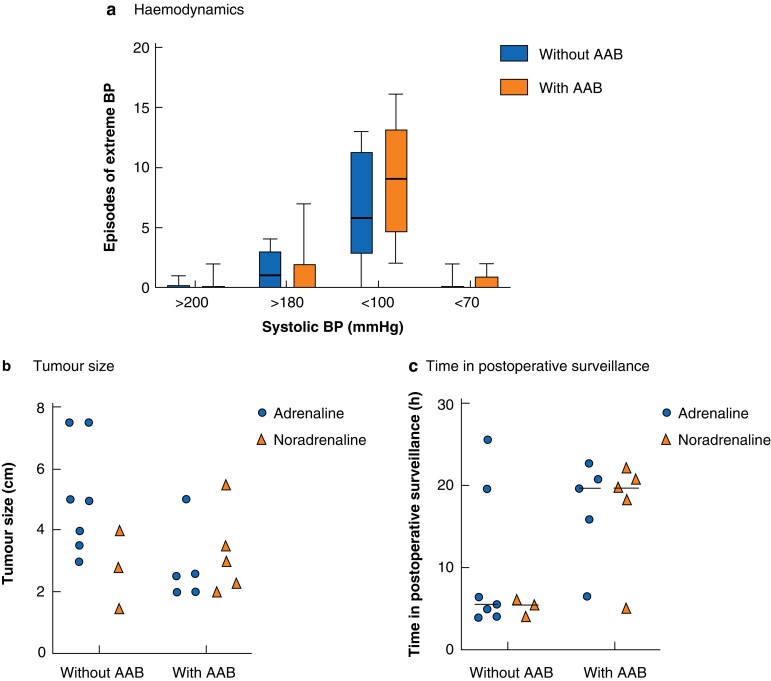
Perioperative haemodynamics, postoperative surveillance time, tumour size, and hormone profile according to pretreatment with α-adrenoceptor blockade **a** Perioperative episodes of extreme BP; median (bold line), i.q.r. (box), and range (error bars) are shown. There was no significant difference in number of episodes with extremely high or low BP between patients operated without α-adrenoceptor blockade (AAB) compared with AAB-pretreated controls. **b** Tumour size in relation to hormone profile. There was no difference in hormone profile or tumour size between groups. **c** Time in postoperative surveillance in relation to hormone profile; median (bar). Patients undergoing phaeochromocytoma surgery without preoperative AAB spent less time in postoperative surveillance, regardless of hormone profile (*P* = 0.035, Mann–Whitney *U* test).

In conclusion, omitting AAB before surgery in patients with phaeochromocytoma does not seem to increase perioperative or postoperative haemodynamic instability, and can shorten both the time in postoperative surveillance units and the overall duration of hospital stay. These data do not, therefore, support current guidelines on phaeochromocytoma management, which recommend treatment with AAB for 7–14 days before surgery to decrease the risk of perioperative haemodynamic instability^1^. An RCT using haemodynamic instability^5^ as endpoint is reasonable and may be feasible.

## Supplementary Material

znac201_Supplementary_Data

## References

[1] Lenders JW, Duh QY, Eisenhofer G, Gimenez-Roqueplo AP, Grebe SKG, Murad MH et al Pheochromocytoma and paraganglioma: an endocrine society clinical practice guideline. J Clin Endocrinol Metab 2014;99:1915–1942 24893135 10.1210/jc.2014-1498

[2] Castinetti F, De Freminville JB, Guerin C, Cornu E, Sarlon G, Amar L. Controversies about the systematic preoperative pharmacological treatment before pheochromocytoma or paraganglioma surgery. Eur J Endocrinol 2022;186:D17–D24 35230260 10.1530/EJE-21-0692

[3] Goldstein RE, O’Neill JA Jr, Holcomb GW III, Morgan WM, Neblett WW, Oates JA et al Clinical experience over 48 years with pheochromocytoma. Ann Surg 1999;229:755–764; discussion 764–756 10363888 10.1097/00000658-199906000-00001 PMC1420821

[4] Groeben H, Walz MK, Nottebaum BJ, Alesina PF, Greenwald A, Schumann R et al International multicentre review of perioperative management and outcome for catecholamine-producing tumours. Br J Surg 2020;107:e170–e178 31903598 10.1002/bjs.11378 PMC8046358

[5] Buitenwerf E, Boekel MF, van der Velde MI, Voogd MF, Kerstens MN, Wietasch GJKG et al The haemodynamic instability score: development and internal validation of a new rating method of intra-operative haemodynamic instability. Eur J Anaesthesiol 2019;36:290–296 30624247 10.1097/EJA.0000000000000941

