# Supplementary material for: α-Adrenoceptor blockers and phaeochromocytoma surgery: outdated combination?
Source: Br J Surg. 2022 Jun 16;109(9):887–8. doi: 10.1093/bjs/znac201 (PMC10364772; doi:10.1093/bjs/znac201)
Supplement: znac201_Supplementary_Data [file znac201_supplementary_data.docx]

**Table S1.** Patient data

|  | Patients (n=10) | Controls (n=10) | *p-value* |
| --- | --- | --- | --- |
| *Background* |  |  |  |
| Age (years) | 56.5 (24-79) | 53 (25-76) | *0.853* |
| Gender (female/male) | 6/4 | 3/7 | *0.28* |
| Hereditary disease |  |  |  |
| - MEN2 | 1 | 1 |  |
| - NF1 | 2 |  |  |
| Comorbidity |  |  |  |
| - Dil. cardiomyopathy | 0 | 1 |  |
| - Atrial fibrillation | 2 | 0 |  |
| - Kidney disease | 0 | 0 |  |
| - Diabetes | 0 | 0 |  |
| Tumour size (mm)  Incidentaloma (yes/no) | 40.0 (15-75)  8/2 | 25.5 (20-55)  6/4 | *0.089*  *0.481* |
| Hormone xULN* | 4.8 (2-12.2) | 5.0 (1.4-13.6) | *0.796* |
| Hormone profile (A/NA) | 7/3 | 5/5 | *0.481* |
| *Perioperative treatment* |  |  |  |
| AAB (n) | 0 | 10 |  |
| phenoxybenzamine (n) | / | 4 |  |
| daily dose (mg) | / | 95 (50-160) |  |
| doxazosin (n) | / | 6 |  |
| daily dose (mg) | / | 16 (8-20) |  |
| Surgical approach (RP/Lap) | 2/8 | 8/2 | *0.023* |
| Infusion of vasodilatorsƗ (n) | 9 | 2 | *0.007* |
| Vasoconstrictive agent needed (n) | 3 | 3 | *1.0* |
| *Postoperative management* |  |  |  |
| Time in postop surveillance unit (h) | 5.5 (4 – 26) | 20.0 (5 – 23) | *0.035* |
| Total inpatient time (days) | 3.5 (2 – 4) | 10.5 (5 – 30) | *0.000* |
| 30 day complications (CD ≥3a) | 0 | 0 |  |

Continuous values presented as median (range). MEN2, multiple endocrine neoplasia type 2; NF1, neurofibromatosis type 1; *xULN “times upper limit of normal” was calculated as the highest measured hormone level divided by the upper limit of normal. **Ɨ**nitroprusside or nitroglycerine; A, adrenaline (epinephrine); NA, noradrenaline (norepinephrine); AAB, alpha-adrenoceptor blockade; RP, retroperitoneoscopic adrenalectomy; Lap, transabdominal laparoscopic adrenalectomy; CD, Clavien-Dindo classification.
